# Taurine-Modified Boehmite Nanoparticles for GFRP Wind Turbine Rotor Blade Fatigue Life Enhancement

**DOI:** 10.3390/ma14226997

**Published:** 2021-11-18

**Authors:** Till Julian Adam, Wibke Exner, Peter Wierach

**Affiliations:** Department of Multifunctional Materials, Institute of Composite Structures and Adaptive Systems, German Aerospace Center (DLR e. V.), Lilienthalplatz 7, 38108 Braunschweig, Germany; Wibke.Exner@dlr.de (W.E.); Peter.Wierach@dlr.de (P.W.)

**Keywords:** fatigue life enhancement, glass fibre-reinforced composites, boehmite, taurine, nanoparticles wind turbine, rotor blade

## Abstract

Advanced nanoparticle-reinforced glass fibre composites represent a promising approach to improving the service life of fatigue-loaded structures such as wind turbine rotor blades. However, processing particle-reinforced resins using advanced infusion techniques is problematic due to, for example, higher viscosity as well as filtering effects. In this work, the effects of boehmite nanoparticles on viscosity, static properties and fatigue life are investigated experimentally. Whereas rheological analysis reveals a significant increase of viscosity in the case of pristine boehmite particles, an additional taurine surface modification of the particles can effectively reduce viscosity increase. As regards mechanical properties, significant improvements of both static as well as fatigue properties are found. The addition of 15 wt.% of boehmite particles increases fatigue life by a maximum of 270% compared to the unmodified fibre-reinforced epoxy. Transmitted light-based investigation of the damage mechanisms shows delayed initiation and smaller growth rates for laminates containing boehmite particles. At the same time, the observed mechanisms and their accumulation along the relative cycle number do not change significantly. In addition, by characterising autonomous heating, the so-called Risitano fatigue limit is determined. The results reveal that with increasing particle content there is an increase in the fatigue limit.

## 1. Introduction

For a successful energy transition, it is necessary to increase the efficiency of energy generation from renewable sources. In the field of wind energy, it is essential to increase the yield per turbine by reducing the turn-off times and increasing the rotor diameter. With the rotor blades among the most fatigue-stressed technical components in terms of fatigue life time, load variability and ambient conditions [1,2], there is a need for stronger and more damage-resistant light-weight materials [3]. In the design process of wind turbine rotor blades, fatigue is one of the most important issues [4]. Therefore, excessive experimental characterisation of wind turbine composite materials [5,6] and rotor blade damage behaviour [5,7,8,9,10,11] has been conducted over the last decades in addition to improving design and fatigue life prediction methods [1,4,12]. An improvement of the fatigue characteristics of the glass fibre-reinforced plastics (GFRP) not only increases the reliability of the rotor blades but also enables their size to be enlarged through a mass-reduced design.

One promising approach to improving the fatigue life of GFRP is the incorporation of nanoparticles [13]. Various studies have already proven the positive effects of fillers on the fatigue life of fibre-reinforced composites. However, the effects depend strongly on the type and amount of the filler, the layup of the fibres and the type of load applied. Reported results range from decreasing fatigue life [14] up to 100-fold extension [15]. Most results show a 3- to 10-fold increase [13,16,17,18,19,20,21]. Some authors have also compared the failure mechanisms of particle-modified and unfilled fibre-reinforced plastics. They have found suppressed matrix cracking and reduced crack growth for the particle-reinforced components. This results in a delayed onset and a reduced level of degradation [18,20,22,23]. The underlying mechanisms depend strongly on the characteristics of the chosen filler. Fenner et al. [16] and Knoll et al. [22], for example, examined graphene and/or carbon nanotubes (CNTs). The carbon fillers increase the energy absorption and change the dominant damage mechanism. Fenner et al. performed fatigue crack growth tests and observed a lower crack growth rate and a higher critical stress intensity factor for the onset of the crack growth. These enhancements are explained by the pull-out of the CNTs and alterations to the morphology of the crack surface. Knoll et al. also took a closer look at the mechanisms of the carbon fillers. By means of fractography, they also found particle-matrix debonding, plastic void growth and nanotube pull-out as toughening mechanisms. Bourchak et al. [23] investigated the effects of single-walled CNTs and graphene nanoplatelets (GNPs) on fatigue of an antisymmetric GFRP laminate and report a 3- (GNPs) and 12-fold (CNTs) increase of fatigue life. In addition to a slower stiffness degradation the characteristic damage state (crack saturation) is delayed and energy dissipation is lower. Completely different characteristics are shown by rubber nanoparticles, which have a low modulus and a more spherical shape. However, their effect on fatigue life is as least as good as that of carbon fillers [15,18,20,21,24,25]. Here the toughening results from the cavitation of the rubber particles, which leads to enhanced plastic-shear deformation. The energy is absorbed by shear banding and void growth in the epoxy [21].

In summary, the use of carbon and rubber fillers for reinforcing fibre-reinforced plastics shows excellent results in increasing fatigue life. However, they are not suitable for wind energy applications. Driven by the cost pressure in the wind industry, rotor blades are manufactured using the out-of-autoclave infusion process, and CNTs and graphene cannot be processed as they are filtered out by the fibres during impregnation. Rubber particles can increase fatigue strength but they reduce the stiffness of the rotor blade. This is different for ceramic fillers which are available as spherical nanoscale particles with a high modulus. There are several publications in the literature that report improvements in fatigue strength by integrating silica or alumina nanoparticles [13,17,18,20,21,25,26]. Tate et al. [13] demonstrate a 3- to 10-fold improvement of fatigue life under tension-tension loading for a GFRP wind energy material containing 6 wt.% of nanosilica particles. Fatigue life time of a nanosilica modified CFRP material investigated by Kamble et al. [27] is 6 to 7 times higher compared to the reference composite. The improvement is attributed to the ability of the particles to disrupt and prolong the propagation of incipient cracks. Munjunatha et al. [20] report a 3 to 4 times longer fatigue life of silica modified glass fibre reinforced epoxies compared to the unmodified material. Under cyclic tension loading, the initiation and growth of interlaminar delamination are observed. For the particle-modified material (10 wt.% silica) the cracking is reduced and the crack densities reached are lower. Reduced cracking results in reduced stiffness degradation. Using scanning electron microscopy (SEM), it is revealed that this effect may be attributed to debonding and subsequent void growth initiated by the particles. However, a meaningful interpretation of the SEM pictures is difficult due to the small size of the particles.

This paper focuses on boehmite nanoparticles which belong to the group of ceramic fillers. As high modulus particles, they not only have the potential to prolong the fatigue life of glass fibre-reinforced plastics. Improvements in the static mechanical performance have already been proven for the chosen particles [28,29]. The flow behaviour of liquid boehmite nanocomposites [30] has also been investigated. It was found that the addition of nanoparticles increases the viscosity to such an extent that infusion processing is no longer possible. Therefore, an additional taurine surface modification of the particles was investigated to positively affect viscosity. In contrast to the pristine particles, taurine-modified boehmite particles were found to result in a significantly smaller increase in viscosity. The different impacts on viscosity are attributed to different particle-resin as well as particle-particle interactions due to the specific surface properties [30,31,32]. Pristine boehmite particles have an increased number of hydroxyl groups that are able to react with the epoxy resin and also promote particle-particle interactions both causing an increase in viscosity. Concerning the surface modification, it is assumed that the hydroxyl groups are blocked by the less polar taurine (undissociated, non-ionic) providing an overall less polar particle surface. The greater difference in the surface energies of particles and matrix results in a lower wetting, less internal friction and consequently lower viscosity. For boehmite particles, Jux [32] also investigated surface modifications with acetic acid, stearic acid, lactid acid, 12-hydroxytearic acid and APTES for different particle weight fractions. It is concluded that non-reactive carboxylic acids with long molecular chains and molecules with free amino groups that have no further chemical functionalities are suitable for the production of low viscosity boehmite-nanocomposites.

In this study, the former results on taurine-modified boehmite nanocomposites [30] are reproduced for a wind energy resin system. The new material is then tested under cyclic tension-compression loading until final failure. In addition, the degradation behaviour and the damage growth of the material are analysed based on digital damage images. Furthermore, the Risitano thermographic approach [33] is used to determine the fatigue limit. All tests are performed with four different particle contents between 0 wt.% and 15 wt.%. For a filler content of 10 wt.% laminates with conventional as well as surface-modified particles are investigated to reveal a possible influence of the surface modification on static and fatigue strength. Comprehensive experimental testing is conducted to show the potential of nanocomposites for fatigue life enhancement of glass fibre composites for wind turbine rotor blades.

## 2. Materials and Methods

### 2.1. Matrix Material Modification

A low viscosity infusion system designed for blades of wind turbines was used. The system consisted of the AIRSTONE 880E epoxy resin and the AIRSTONE 886H amine hardener (Olin Germany, Stade, Germany). As the nanoscale matrix additive, the commercially available boehmite nanoparticles DISPERAL HP14 (Sasol Germany GmbH, Hamburg, Germany) were chosen. The particles had a cuboid shape and a crystallite size of 14 nm. The boehmite nanoparticles were used with a taurine modification (HP14T) on their surface and also without any modification (HP14). The spray-dried powder was mixed into the epoxy resin with a high filler content of 30 wt.% and subsequently dispersed on a three-roll mill (Exakt Advanced Technologies GmbH, 80E, Norderstedt, Germany) to ensure fine and homogenous particle distribution. During this process, the size of the gaps between the rolls was successively decreased, which increased the shear forces within the gaps. The dispersing was continued until no further particle size reduction was observed by means of a disc centrifuge (CPS Disc Centrifuge, CPS Instruments Inc., Prairieville, LA, USA). The final mean particle size was 124 nm for the unmodified and 170 nm for the taurine-modified particles. This means that the particles have a fine and homogenous distribution, but the nanocomposites also form smaller agglomerates. Finally, the dispersion was diluted with epoxy resin to adjust the filler content and the hardener was added in a weight ratio of 100:31. In this study, the examined weight ratios of the particles were 0 wt.%, 5 wt.%, 10 wt.% and 15 wt.% in regard to the overall nanocomposite. In addition, the viscosity of the nanocomposite with 10 wt.% particles was analysed using a plate-plate rheometer (Gemini HR nano, Malvern Instruments GmbH, Herrenberg, Germany) at 70 °C. The measurement was performed with a plate diameter of 40 mm, a gap size of 1 mm and a rotational speed of 1 s^−1^.

### 2.2. Laminate Fabrication

An E-glass fibre fabric (U-E-1200 g/m^2^–1300 mm, Saertex GmbH, Saerbeck, Germany) was used to manufacture the fibre-reinforced composites. Here, the unidirectional fabric was stacked in a [+45, −45]_s_ sequence. Before impregnation, the liquid nanocomposite was degassed at 10 mbar for 10 min to remove any entrapped air. The fibre-reinforced nanocomposite was manufactured by a vacuum-assisted resin infusion process in an autoclave. Autoclave processing is not an established practice for rotor blades, as they are commonly manufactured out-of-autoclave for reasons of economy. However, the autoclave process allows a more homogeneous fibre volume content and fewer voids. Infusion was performed by peripheral injection and the resin was distributed by a net bleeder. The final impregnation was performed in the thickness direction at a temperature of 70 °C. Curing was conducted at 80 °C for 6 h. Peel ply was used on both sides of the stacking to facilitate demoulding.

### 2.3. Specimen Design and Fabrication

**Figure 1:**
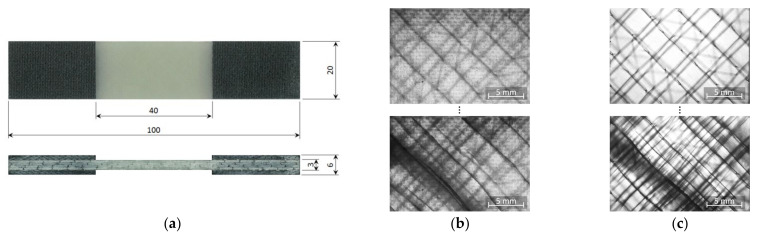
(**a**) Overview of the coupon specimen geometry for quasi-static and fatigue testing (all dimensions in mm); (**b**) transmitted light images of the regular laminate with peel-ply artefacts (undamaged/damaged); (**c**) transmitted light images of the polished laminate (undamaged/damaged).

Two parameters, the maximum force of the ZwickRoell GmbH & Co. KG (Ulm, Germany) LTM 10 electrodynamic testing machine ($F_{\max}=10\;\mathrm{kN}$) and the chosen cyclic load ratio (R=−1) crucially determine specimen geometry. Due to the low maximum force and the given laminate thickness, the specimen width had to be limited to w=20 mm. As buckling is an issue when testing standard coupon specimens (typical free length l>100 mm, cp. [34]) under cyclic tension-compression loads, anti-buckling jigs [35] are usually used. However, anti-buckling jigs are known to cause frictional heating and impede optical online damage monitoring. A much shorter free specimen length of l=40 mm was therefore chosen to prevent undesired buckling. The stability of the specimens was assessed by determining the normal and buckling strains by means of strain gauges. For quasi-static compression loads up to F=−5 kN maximum, the bending surface strain ($\varepsilon_{b,-5\mathrm{kN}}=-0.013\%$) was only about 1.4% of the normal strain ($\varepsilon_{n,-5\mathrm{kN}}=-0.94\%$). Furthermore, a high-speed camera was used to qualitatively characterise failure under tension-compression fatigue loading. Deformation behaviour under fatigue loads was found to be clearly dominated by normal strains without visible buckling prior to final rupture. The final specimen geometry is depicted in Figure 1a. After bonding the end tab laminates by means of a hot press, specimens were cut with a water-cooled diamond blade saw. Whereas this type of specimen was used for the regular tests, a small set of surface-polished specimens was prepared for investigating damage development. For this purpose, the laminate was wet-grinded and polished after the removal of the peel ply, providing a much higher optical transparency. Only the thin resin pattern film was removed, without causing damage to the glass fibres. Specimen cutting and tabbing was identical to the regular specimens. An exemplary comparison of transmitted light images of the regular and the polished laminate is given in Figure 1b,c. Whereas surface-near cracks are quite distinctive in both images, inner ply cracks and also the sewing yarn cannot be identified in the unpolished laminate. Delamination is visible but its contours are blurred. Although damage mechanisms can be identified much better in the polished laminate, through-the-thickness visibility is limited due to the optical properties of the matrix material and the specimen thickness.

### 2.4. Experimental Set-Up and Testing Procedures

All quasi-static and fatigue tests were conducted by means of a Zwick LTM 10 electrodynamic testing machine with a dynamic force range of $F_{a}=\pm 10\;\mathrm{kN}$. Specimens were clamped in place with pneumatically actuated wedge grips. The Zwick testXpert II (static tests) and Zwick testXpert R (dynamic tests) testing software was used for configuring the test procedures, test control and data acquisition (cylinder displacement and force).

**Figure 2:**
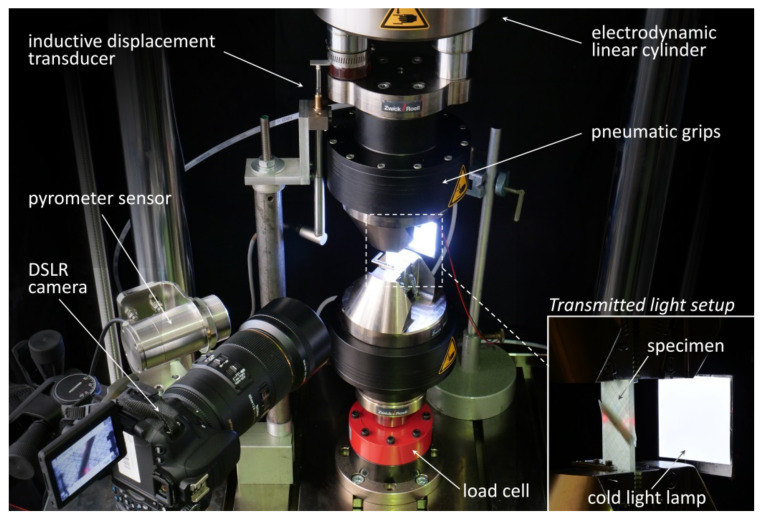
Overview of the experimental set-up: Zwick LTM10 electrodynamic testing machine with an additional transmitted light set-up and pyrometer-based specimen temperature measurement.

Furthermore, a high-performance Micro Epsilon CTL-CF2-C3 pyrometer sensor (Ortenburg, Germany) with laser measuring field marking was used to monitor specimen surface temperature in the free specimen range. By adjusting the circular measuring field to almost the full specimen width, a representative average surface temperature was detected. Temperature monitoring is important for two reasons. Firstly, specimen heating has to be controlled to prevent overheating and temperature-induced failure. This was done by limiting the test frequency and by active cooling with oil-free compressed air. Secondly, temperature data was used to estimate the materials fatigue limit by means of the Risitano thermographic method [33]. With regard to optical damage monitoring, a transmitted light system consisting of a Canon EOS 650D DSLR camera (Tokyo, Japan) equipped with a SIGMA 105 mm macro lens (Kawasaki, Japan) and a cold light lamp on the opposite side of the specimen was set up. By automatically triggering the transmitted light imaging (TLI) during quasi-static characterisation steps, micro- and macroscopic fatigue damage development (matrix micro-cracking, interlaminar delamination) could be captured at discrete cycle numbers. To prevent ambient light effects, the whole set-up was darkened by means of black cotton molton. Consistent image exposure within a single fatigue test and also within a test series is of high relevance for automatic damage detection based on digital image analysis. An overview of the experimental setup is depicted in Figure 2.

#### 2.4.1. Static Tests

Quasi-static tests were primarily conducted for the purpose of defining the load levels for the fatigue tests. Furthermore, the effect of nano-modification on static strength was investigated. The European test standards DIN EN ISO 527-4 (tensile properties) and DIN EN 6031 (shear properties: ±45° tensile test) could not be strictly followed as regards the specimen geometry and strain measurement. Instead of using strain gauges (SG) on every specimen, strains were calculated using the displacement signal of the testing machine and an SG-displacement correlation. Due to this simplification, the resulting elastic moduli and rupture strains have to be understood as approximations. The nominal tensile stresses calculated by means of Equation (1), however, are accurate. According to DIN EN 6031, the in-plane shear strength is $\tau_{\mathrm{xy},u}=1/2\cdot \sigma_{x,u}$. However, for the sake of clarity, the authors prefer using the nominal stress $\sigma_{x,u}$. Tests were conducted at room temperature and with a displacement rate of 1.0 mm/min.

(1) $$\sigma_{x,u}=\frac{P_{\max}}{A_{m}}$$

#### 2.4.2. Fatigue Tests

All fatigue tests were cyclic tension-compression tests with a load ratio of R=−1. Six cyclic load levels with load amplitudes between 75% and 30% of the static strength were tested. Tests had to be conducted at different frequencies to prevent specimen overheating at the higher load levels (1 Hz) and to shorten test durations at medium (3 Hz) and low load levels (6 Hz) with failure in the high cycle fatigue range.

**Figure 3:**
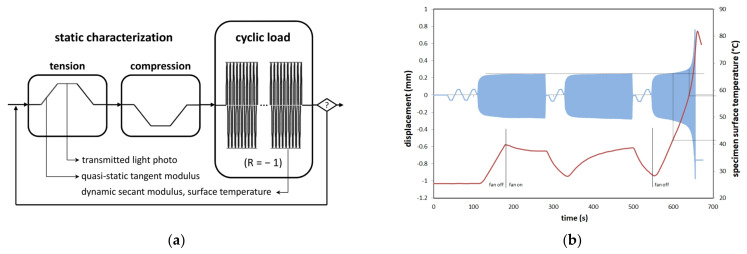
(**a**) Interrupted fatigue test sequence containing static characterisation steps for transmitted light monitoring and tangent modulus determination, measurement of surface temperature and dynamic secant modulus during the fatigue loading blocks; (**b**) Exemplary plot of specimen surface temperature development during cyclic loading, effect of air-cooling and temperature-induced thermo-mechanical failure without cooling.

Several damage monitoring techniques (online transmitted light imaging, stiffness degradation characterisation, temperature monitoring) were applied. Although transmitted light imaging is not suitable for investigating particle-damage interactions on the nano-scale, it proved to be a powerful method of investigating the effect of nano-modification on micro- and macro-scaled damage development. Regarding stiffness characterisation, both dynamic and quasi-static moduli were determined. As the cyclic tests had to be interrupted for transmitted light imaging and quasi-static characterisations, a typical test sequence of alternating characterisation and fatigue steps was used (Figure 3a).

Characterisation intervals were load level-dependent and ranged between 100 load cycles at load level 2 and 50,000 cycles at load level 6. The characterisation ramp loads were set to ±1 kN, sufficient for modulus determination and small enough to cause no further damage. Due to the angle-ply configuration and resulting shear stresses, specimen heating was observed to be an issue. Figure 3b illustrates specimen surface temperature development during cyclic loading of an exemplary pre-test. With air cooling (fan on), specimen heating could be contained and steady-state conduction was reached at a temperature of approx. $40\;{\;}^{{}^{\circ}}C$. Without air cooling (fan off), specimen overheating and subsequent thermo-mechanical failure was observed when the temperature rose above $57{\;}^{{}^{\circ}}C$. To prevent any undesired temperature effects, specimen cooling was improved by means of oil-free pressurised air. Except for the temperature rise caused by final failure, temperatures could effectively be kept below $30{\;}^{{}^{\circ}}C$ during the fatigue tests. Tests were stopped automatically after specimen rupture when elongation reached 1 mm (loss of specimen integrity, but no catastrophic specimen destruction).

As regards the statistical analysis of the gained S-N data, the common linear relationship between the logarithm of stress (S) and cycles to failure (N) was used (Equation (2)). Furthermore, the 95% prediction limits $N_{P\%}^{\pm }$ (Equation (3)) were calculated according to Schneider and Maddox [36].

(2) log N=logA−m logS

(3) $$\log N_{P\%}^{\pm }=\left(\log A+m\log S\right)\;\pm \;t\hat{\sigma }\sqrt{1+\frac{1}{n}+\frac{{\left(\log S-\bar{\log S}\right)}^{2}}{\sum_{i=1}^{n}{\left(\log S_{i}-\bar{\log S}\right)}^{2}}}$$

#### 2.4.3. Thermographic Fatigue Limit Estimation

In addition to the regular fatigue test series, the Risitano thermographic approach [33] for rapid fatigue limit determination was applied. The method was originally developed and validated for metals [37,38] but it has also been successfully applied to fibre-reinforced laminates [39,40,41,42,43]. The main advantage of this method is that the fatigue limit can be determined by means of a single specimen and in a sequence of cyclic heating tests lasting a few hours. The basic assumption of the method is that heat dissipation due to intrinsic energy dissipative mechanisms (viscoelastic self-heating, micro-damage initiation, micro-cracking, delamination, fibre fracture) increases drastically when the material is stressed above its fatigue limit. By means of thermography (here a pyrometer is used) the stabilisation temperature of a specimen is determined for increasing stress levels until failure. By correlating stabilisation temperature and load amplitude, the fatigue limit can then be identified. In this study, the method is used to investigate whether adding nanoparticles changes the Risitano fatigue limit and whether this is in line with the results of the conventional static and fatigue test series.

### 2.5. Digital Damage Image Analysis

**Figure 4:**
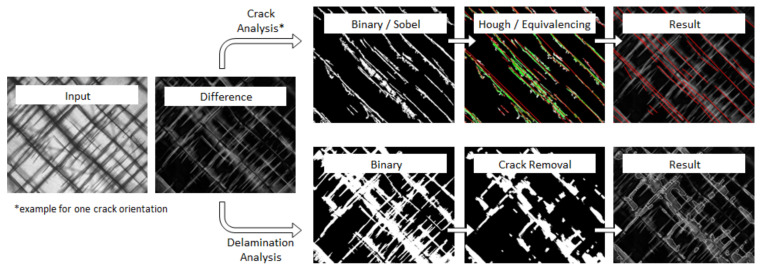
Illustration of the digital image analysis procedure used for damage content quantification.

During the fatigue tests, an automatically triggered transmitted light system was used to capture the damage states at distinct cycle numbers. As the system is based on a high-resolution DSLR camera, the images provide good insights into damage behaviour. In addition to characterising the fatigue damage development in the reference material, the impact of nano-modification on the fatigue mechanisms (delamination and matrix micro-cracking) was investigated. In order to quantify the damage contents, a digital image analysis tool programmed in Python 2.7.10.0 was used. An overview of the damage analysis procedure is depicted in Figure 4 for an exemplary damage image.

For the sake of clarity, only the upper half of the specimen’s test section is shown. Prior to the digital damage analysis, all raw images belonging to one test series are processed (sharpening, contrast improvement) with Adobe Lightroom and aligned using Adobe Photoshop. In the first step of the automatic damage evaluation, difference images between the first (no damages) and all subsequent images (increasing damage content) are created. During this process, all static objects (e.g., the sewing yarn) are removed.

Crack analysis is based on binarisation, Sobel filtering and Hough transformation. Sobel filtering is used to extract all equally-oriented cracks. Hough line detection is followed by an equivalence algorithm merging multiple detections in one representative line. The length of all single cracks $l_{\mathrm{crack},i}$ are then summed up and divided by the evaluation window area according to Equation (4). As damage state analysis revealed that crack density developed similarly in both laminate directions ($+45^{{}^{\circ}},-45^{{}^{\circ}}$), an average crack density (cp. [20]) was defined (Equation (5)).

(4) $$\rho_{\alpha }=\frac{1}{A_{\mathrm{eval}}}\left(\sum_{i=0}^{m}l_{\mathrm{crack},i}\right)$$

(5) $$\rho =\frac{1}{2}\cdot \;\left(\rho_{+45}+\rho_{-45}\right)$$

As regards the delamination analysis, difference images are again transformed to binary images using a much lower binarisation threshold. All cracks are then detected and removed. The remaining objects represent the delaminated area. Based on the total delamination area, the delamination fraction $H_{\mathrm{del}}$ is calculated.

(6) $$H_{\mathrm{del}}=\frac{A_{\mathrm{del}}}{A_{\mathrm{eval}}}$$

## 3. Results and Discussion

### 3.1. Rheological Properties

**Figure 5:**
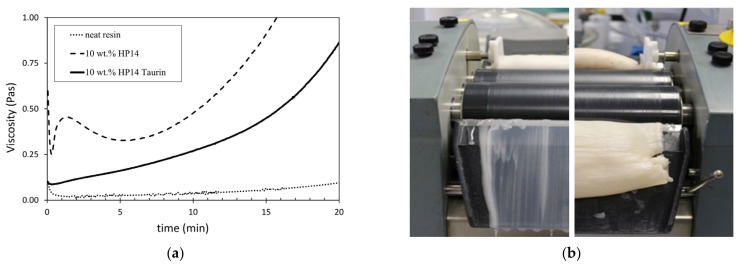
(**a**) Viscosity over time of the neat resin and the nanocomposites with 10 wt.% HP14 and HP14 taurine analysed by a plate-plate rheometer at 70 °C; (**b**) Flow behaviour of the masterbatch during dispersion by a three-roll mill: epoxy resin with 30 wt.% HP14 taurine (**left**), epoxy resin with 30 wt.% HP14 (**right**).

Isothermal rheological analyses of the neat resin and the two nanocomposites with 10 wt.% of unmodified and taurine-modified particles are conducted at a temperature of $70{\;}^{{}^{\circ}}C$. This elevated temperature equals the infusion temperature used for laminate fabrication. Figure 5a shows the rheometric results. It can be seen that the addition of the unmodified particles causes a significant rise of viscosity compared to the reference resin, which is a well-known effect. Fluctuations in viscosity at the beginning of the measurement result from the rising temperature and the thixotropic flow behaviour of the nanocomposite, as no pre-heating and pre-shearing of the samples was carried out.

Viscosity increases over time, which is a result of the progressive curing of the resin. With a starting viscosity of around 0.3 Pas, the nanocomposite containing the unmodified particles is problematic for infusion. The flow limit is generally assumed to be 0.5 Pas [44]. Contrary to this, the nanocomposite with taurine-modified particles starts with a much lower viscosity of approx. 0.1 Pas, which is suitable for impregnation processes. Although the viscosity of the taurine modified nanocomposite is higher than the viscosity of the neat resin, achieved viscosity reduction compared to the unmodified nanocomposite is significant. The different effects of both particles become obvious in Figure 5b. During dispersion of the two masterbatches with 30 wt.% particles on the three-roll mill, the nanocomposite with taurine-modified particles ran down the removal plate. In contrast, the nanocomposite with unmodified particles was much more viscose and did not run at all.

### 3.2. Static Tensile Properties

Starting with the reference laminate containing no nanoparticulates (0 wt.% (Ref.)), particulate contents of 5 wt.%, 10 wt.%, and 15 wt.% were investigated. An additional surface treatment of the particulates with taurine (TM: Taurine Modification) was used to significantly reduce resin viscosity and to improve the impregnation of the glass fibre material. The 10 wt.% modification was tested with and without taurine surface modification to assess its potential effects on the static and fatigue properties.

**Figure 6:**
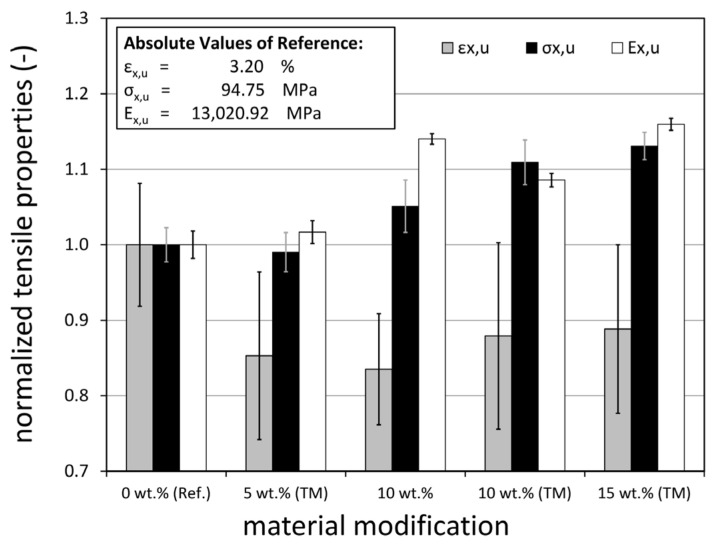
Normalised results of the quasi-static tensile tests of all laminate modifications. All strains to rupture, ultimate stresses and elastic moduli constitute average results of at least six specimens.

The main results of the quasi-static (q.s.) tensile tests of all five material modifications are depicted in Table 1 and Figure 6. Compared to the reference material (0 wt.%), nanoparticle reinforcement has significant effects on the elastic modulus, rupture strain and tensile strength.

On the normalised scale (Figure 6), the value 1 represents the properties of the unmodified reference material. With regard to the effects of particle modification, it can be seen that adding a boehmite content of 5 wt.% does not significantly change the tensile properties. In fact, the tensile strength and longitudinal modulus remain more or less unchanged. In contrast, there is a 15% decrease in the strain to rupture which likewise occurs for all higher particle contents. Derived strains to rupture, however, are of limited significance as they are based on the internal displacement sensors of the testing machine and show high scattering. This also holds true for the elastic moduli. The results of the tensile stresses are much more consistent. It can be seen that both increasing particle content and modifying the particle surfaces with taurine improves tensile strengths compared to the reference laminate. A maximum increase of about 13% is observed for the highest taurine-modified particle content (15 wt.% (TM)). Comparison of the results of the two 10 wt.% modifications reveals that taurine modification results in an additional 5.5% increase in strength. This, however, does not necessarily indicate that the modification itself improves the laminate strength, for example, due to better matrix-particle bonding. It is more likely that the particles are distributed more homogeneously due to the lower viscosity of the resin. Likewise, the lower viscosity might simply result in better impregnation of the glass fibre material and a better laminate quality in terms of manufacturing defect content.

### 3.3. Fatigue Behaviour

Comprehensive cyclic tests of the GFRP angle-ply configuration with different boehmite particulate-based matrix modifications were conducted. In addition to the conventional test specimens, a limited number of surface-polished specimens (P) with improved damage visibility were tested at two load fatigue levels.

An overview of all matrix modifications, with the most important test parameters and results, is shown in Table 2. Fatigue tests (R=−1) were conducted at six different load levels (LL: 1–6) with stress amplitudes ($\sigma_{a}$) in the range between 74% and 30% of the static ultimate strength ($\sigma_{\mathrm{ult}}$). As described in the method section, test frequency and cycles per fatigue load block were chosen in dependence on the load level. In addition to the relationship between stress amplitude and fatigue life cycles (S-N), stiffness degradation and damage growth were investigated. Optical damage investigations were conducted using polished specimens. Furthermore, two cyclic test series of three 0 wt.% (Ref.) and three 15 wt.% (TM) specimens were conducted applying the Risitano thermographic fatigue limit determination method [33].

#### 3.3.1. Fatigue Life

**Figure 7:**
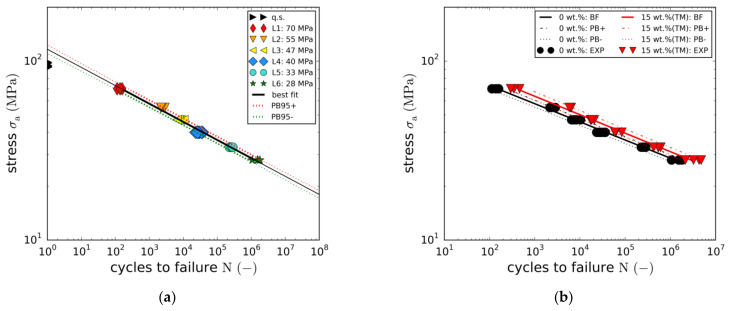
(**a**) S-N-curve of the reference material; (**b**) S-N-curves of reference GFRP and with 15 wt.% (TM) taurine-modified boehmite nanoparticles.

The fatigue results of the reference material (0 wt.% (Ref.)) in terms of stress amplitude vs. the number of cycles to failure are depicted in Figure 7. Whereas average fatigue life is 137  load cycles at the highest load amplitude (LL1: 70 MPa), failure occurs at about $1.48\;\cdot 10^{6}$ cycles at the lowest load level tested (LL6: 28 MPa.) The full set of S-N data points is well-represented by a power law or the linear relationship of the logarithmic values (Equation (2)). Due to the noticeable low scattering of the load level-specific failure cycles, the prediction band of the regression (Equation (3)) is rather narrow. In fact, the average percentage standard deviation of the cycles to failure is only about 14%. This is probably due to the high precision of the testing machine and the quality of the laminate material. Extrapolation of the S-N relationship towards lower failure cycles reveals that the quasi-static data points are not matched. This is typical for laminates containing angled plies, as damage behaviour is different under quasi-static and cyclic loads.

The second laminate tested was the 15 wt.% (TM) modification with the maximum boehmite particle content of 15 wt.%. Like the reference laminate, it was tested at all six different load levels. The resulting S-N curve is compared to the reference curve in Figure 7b. The figure reveals that adding 15 wt.% of taurine-modified boehmite particles provides significant fatigue life enhancements at all load levels. Average fatigue life enhancement is about 240%. Average fatigue strength increase over all load levels is about 10%. Again, scattering is remarkably low.

**Figure 8:**
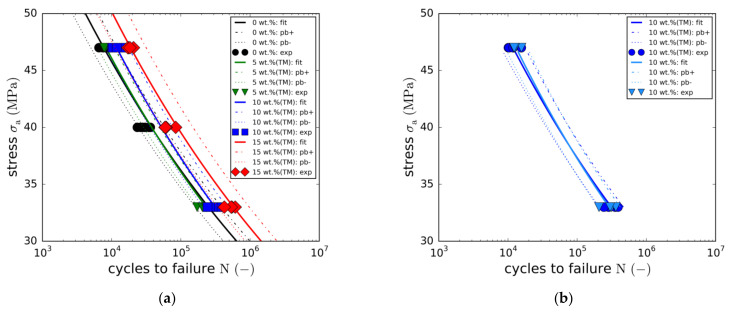
(**a**) Comparison of reference GFRP with 5 wt.%, 10 wt.% and 15 wt.% boehmite nanoparticles; (**b**) Effect of taurine surface modification on fatigue life.

With regard to the other modifications (5 wt.% (TM), 10 wt.% and 10 wt.% (TM)), only two load levels (LL3 and LL5) were tested in order to reduce the overall effort. The resulting S-N curves are compared to the full S-N curves in Figure 8a. As in the case of the static strength, no effect on fatigue life was found for the 5 wt.% (TM) modification.

**Figure 9:**
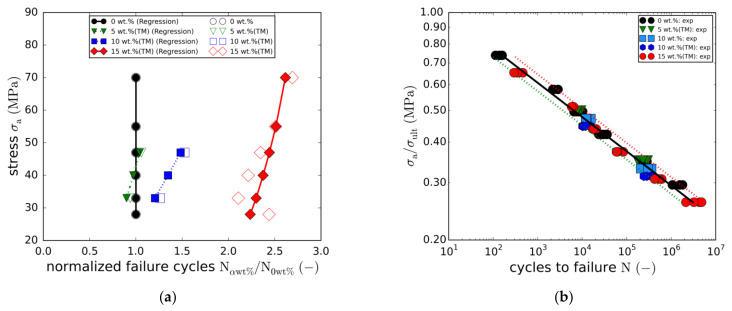
(**a**) Fatigue life multiplication factors in dependence of the load level; (**b**) Master S-N curve.

In fact, the resulting S-N curve is quasi-identical to the reference curve. In contrast, adding a particle content of 10 wt.% results in an average fatigue life enhancement of about 140%. An overview of the detailed load level-specific fatigue life enhancements is depicted in Figure 9a, showing the stress amplitudes plotted against the normalized failure cycles. Whereas the filled markers represent the fitted S-N curves, the unfilled points depict the load level averages of the experimental data points. In all cases, fatigue life enhancement is not constant over stress amplitude. In fact, a linear dependency can be seen, with the fatigue life enhancement increasing with stress amplitude. Although this observation could indicate greater effectiveness of the toughening mechanisms of the nanoparticles at higher stress levels, this could not be completely clarified in this study.

To investigate the potential effect of the taurine modification, equal particle contents of 10 wt.% were tested with and without taurine surface modification. The comparison of both S-N curves (Figure 8b) does not reveal any significant changes in fatigue life. Both curves are nearly identical. This observation is not in line with the results of the quasi-static tensile tests, where the taurine modification was found to cause an increase in the static strength. However, due to the small number of both quasi-static and fatigue data points, this observation may not allow any reasonable conclusions to be drawn.

Tensile strength-based normalisation of the fatigue strengths indicates a proportionality of both properties. This holds true for all modifications with the correlation being most accurate for the reference laminate and the 15 wt.% (TM) modification. Plotting the normalised stress amplitude against cycles to failure reveals that all five cases are roughly represented by means of the single master curve described by Equation (7).

(7) $$\log\;N=\log A-m\;\log\left(\frac{\sigma_{a}}{\sigma_{\mathrm{ult}}}\right)\;\mathrm{with}\;A=8.837,\;m\;=\;9.504$$

Concerning the overall quality and significance of the S-N test results, several observations underlining result validity can be made. Data scattering is rather low which is a result of the high laminate quality, adequate specimen preparation and measurement as well and test conduction with repeated accuracy. Furthermore, with all data points well represented by the regression curve, there are no signs for strain-rate or frequency effects in the case of load levels 1 and 6 which are tested at different test frequencies. This also holds true for temperature, which was monitored and increased not more than 10 °C (except for the final stage of fatigue life) as recommended in ISO13003 [45]. During the static characterization steps, specimens cooled down slightly. This, however, is not assumed to have a significant impact on cycles to fatigue. Additionally, as load-level specific testing sequences were the same for all material configurations, comparative results such as the fatigue life enhancement would not be affected.

#### 3.3.2. Degradation Behaviour

**Figure 10:**
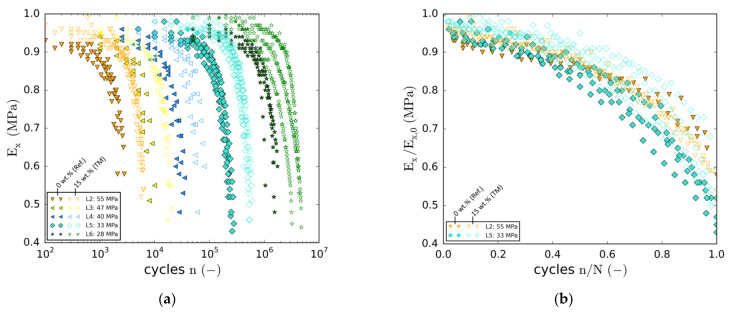
(**a**) Normalised tangent modulus against cycle number (all load levels); (**b**) Normalised modulus against normalised cycle number (two representative load levels).

During the fatigue tests, periodic quasi-static characterisation steps were conducted automatically to monitor the change in longitudinal specimen stiffness $E_{x}$ with increasing cycle number. The results of both, the reference laminate (filled markers) as well as the modification with the highest particle content (15 wt.% (TM), unfilled markers) are depicted in Figure 10a. Each data point represents a quasi-static characterisation test. In the case of the higher load levels (e.g., LL2), stiffness degradation sets in directly at the beginning (not captured due to characterisation interval size). At the time of the first characterisation step, a stiffness loss of about 10% is observed. Due to the strong dependency on the load level, initiation of damage-related stiffness degradation shifts towards higher cycle numbers at lower load levels.

In the case of the lowest load level, no significant loss of stiffness is observed before 50,000 cycles. With regard to the particle-modified specimens, degradation sets in later at all load levels. Thus, for a distinct cycle number, residual stiffnesses are higher in most cases. As regards residual stiffness shortly before final failure, two tendencies can be seen. Firstly, pre-rupture stiffness decreases with load level. Secondly, the majority of all specimens containing nanoparticles reach similar or lower stiffnesses shortly before final failure (except for LL5). Overall, specimen failure occurs at stiffnesses in a range between 40% and 50% of the initial stiffness values.

In Figure 10b, two exemplary and representative load levels (L2 and L5) are chosen to illustrate relative differences in degradation behaviour by means of fatigue life normalisation. Except for slight differences, the degradation behaviour of both laminates is rather similar. At both load levels, initial stiffness degradation (<20% of fatigue life) is slightly more progressive in the case of the particle-free reference laminate. Whereas subsequent degradation until final failure is very similar in the case of the higher load level, degradation of the 15 wt.% (TM) laminate is delayed at the lower load level. In contrast, towards the end of the fatigue life, degradation seems to accelerate for the laminates containing particles. In consideration of the data scattering, however, no distinct conclusions can be drawn.

With regard to the overall degradation behaviour, it has to be pointed out that none of the specimens showed the typical three-staged degradation behaviour known from angle-ply or multiaxial laminates [46,47,48]. In fact, the different stages of fatigue can hardly be identified. After the onset of degradation during the first 20% of fatigue life, stiffness degrades more or less progressively until final failure. Except for the very last cycles, there is no region of distinctively accelerated stiffness degradation between 80% and 100% of fatigue life.

#### 3.3.3. Damage Growth

**Figure 11:**
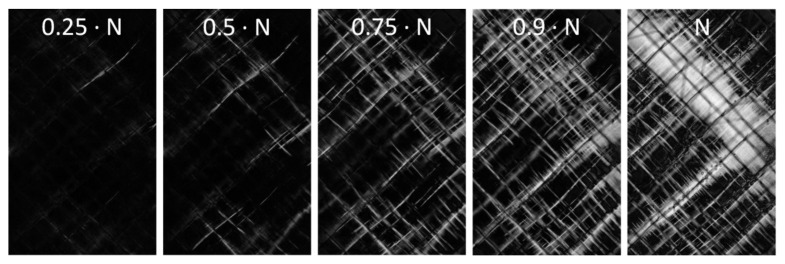
Exemplary transmitted light picture series (difference images) from 25% to 100% of cycles to failure N (0 wt.% (Ref), load level 5).

Regarding the angle-ply laminate under alternating tension-compression load, fibre-parallel matrix cracking and delamination are found to be the dominating damage mechanisms. Damage evolution of an exemplary reference specimen is depicted in Figure 11 by means of transmitted light difference images. The image sequence reveals that damage growth mainly takes place during the second half of fatigue life. After passing 25% of the cycles to failure (N), there are only a few matrix cracks visible in the outer ply. Crack initiation was found to occur in the undisturbed laminate away from the free edges. Inner ply cracking was observed to occur at the same time as or shortly after outer ply crack initiation, leading to a comparable cracking state of both ply orientations at 50% of N. First delamination of the +45° and −45° plies occurs between 50% and 75% of N. Comparison of the images taken at 75% and 90% reveals that crack saturation has not been reached at this point. In fact, cracking proceeds until final failure (cp. rupture image at N cycles). Although neither outer nor inner ply cracking reaches saturation, delamination is the predominating damage mechanism between 90% of N and final failure (N).

**Figure 12:**
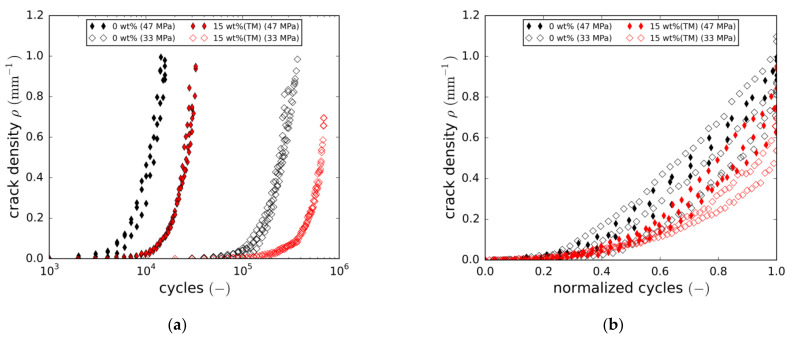
(**a**) Crack density vs. number of cycles, crack density averaged over both cracking directions; (**b**) Crack density vs. number of normalised cycles, crack density averaged over both cracking directions.

**Figure 13:**
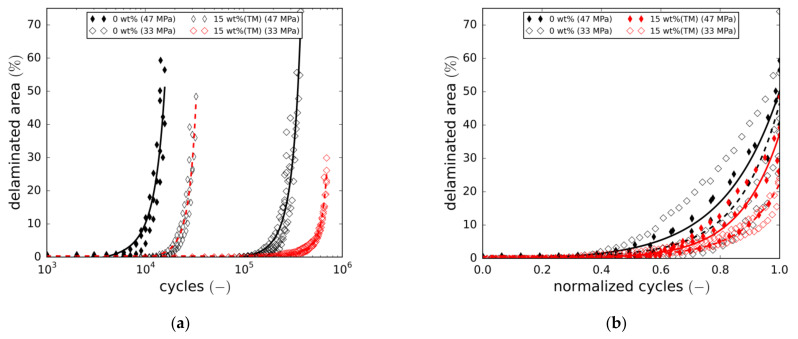
(**a**) Delaminated area fraction vs. number of normalised cycles; (**b**) Correlation of crack density and delamination.

Damage content evolution was evaluated by means of the digital damage image analysis tool presented in Section 2.5. Damage analysis revealed the crack densities of both ply directions to be very similar at all times from initiation to final failure. Thus, a crack density average of both orientations was calculated using Equation (5). Figure 12a shows the development of the crack density for both the reference laminate and the laminate containing 15 wt.% of taurine-modified boehmite particles tested at two load levels. Overall, the increase in crack density reflects the decrease in stiffness in all cases. Adding nanoparticles leads to a significant delay in crack initiation. Furthermore, cracking rates are lower at all times. To reveal relative differences in cracking behaviour, the cycle number is normalised in Figure 12b. At both load levels, the crack growth curves are slightly shifted to higher normalised cycles, implying that the particles cause a change in cracking behaviour. However, closer inspection of the overall crack states, with special attention given to individual crack length development, did not reveal major differences in the cracking mechanism. Similarly to crack density, the fraction of the total delaminated area is plotted in Figure 13a (absolute cycles) and Figure 13b (normalised cycles). Again, a delay in damage initiation and slower progression of the damage are observed.

In addition, final failure seems to occur at lower damage contents (both crack density and delaminated area) in the case of the 15 wt.% (TM) modification. This, in fact, is more pronounced at the lower load level and is also in line with the results discussed in Section 3.3.1. (Figure 9a). The underlying reasons for final failure to occur at lower damage contents could not be clarified in this study. Fatigue life normalisation again reveals a slight right shift of the damage growth curves towards higher relative cycle numbers.

**Figure 14:**
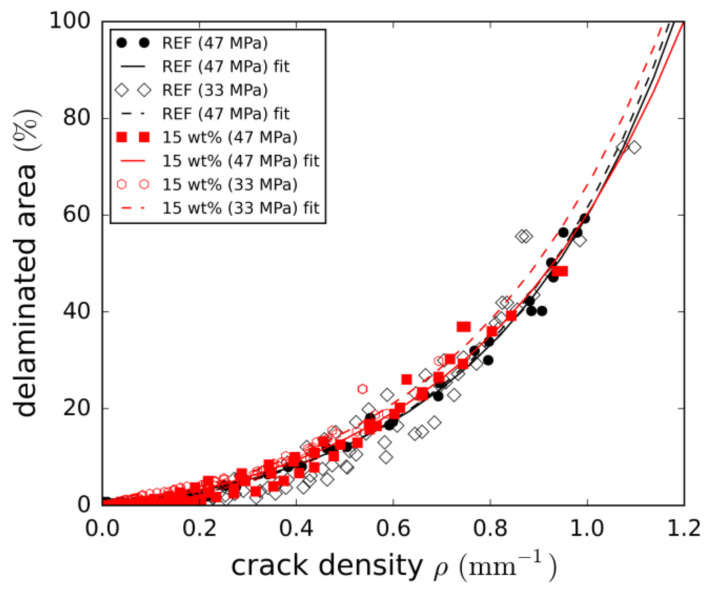
Correlation of crack density and delamination.

The relationship of both damage mechanisms is illustrated in Figure 14 by correlating delaminated areas and crack densities. From this, two conclusions can be drawn. Firstly, the progressive increase of delamination confirms that delamination growth overweighs cracking towards the end of fatigue life. Secondly, as the relationship is more or less independent of both load level and particle content, it can be concluded that both parameters impact the damage mechanisms equally.

All in all, the investigation of the damage parameters for the two-particle contents did not reveal major changes in the damage mechanisms. Both cracking and delamination are simply delayed and have lower growth rates, leading to significant fatigue life enhancements.

#### 3.3.4. Fatigue Limit

To further investigate fatigue life enhancement resulting from the addition of boehmite nanoparticles, the so-called Risitano thermographic approach (Section 2.4.3) was employed. Again, investigations focus on the reference laminate and the modification with the maximum particle content (15 wt.% (T)) to reduce testing effort. Within the additional fatigue tests series, load-dependent specimen heating was captured for three specimens per configuration. Then by correlating stabilisation temperature and load amplitude, the Risitano fatigue limits (RFL) of both configurations were identified.

**Figure 15:**
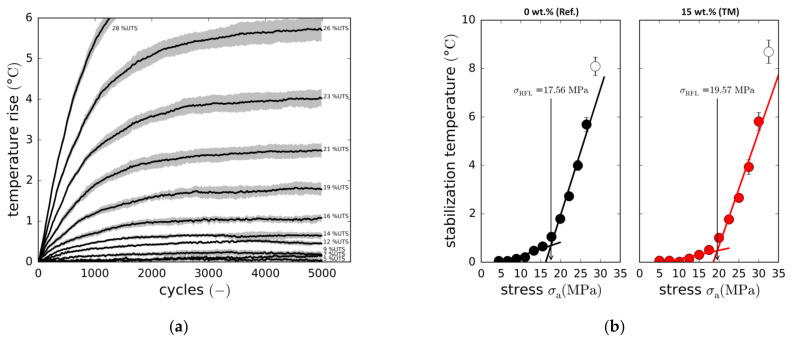
(**a**) Temperature rise of the reference laminate with different relative loads (% UTS), averages of three specimens with standard deviations; (**b**) Stationary temperatures against cyclic load, Risitano fatigue limit (RFL) determination for the reference laminate and the 15 wt.% (TM) boehmite nanoparticle modification.

Figure 15a exemplarily depicts the reference laminate’s (0 wt.%) load-dependent increase in specimen surface temperature within the first 5000 cycles. The temperatures are average temperatures of three specimens. For relative load amplitudes below 26% of the static strength (UTS), temperature stabilises in the range between 2000 and 4000 load cycles. With higher loads (see 28%UTS), temperature equilibrium is not reached, as specimen overheating causes early thermo-mechanical specimen failure. Based on the stabilisation temperatures (temperature averages between 3000 and 5000 cycles), the fatigue limits are identified as depicted in Figure 15b. Adding 15 wt.% of nanoparticles apparently increases the RFL from 17.6 MPa to 19.6 MPa. Although this limit is far below the lowest load amplitude tested in the experiments (L6: 28 MPa), this result clarifies the way the S-N curve changes in the case of the nanoparticle-toughened material. With both the UTS (+13%) and the RFL (+11%) showing similar increases, the S-N curve is shifted to higher stress amplitudes (upwards). However, both the experimental proof of the fatigue limit (high experimental effort and costs) as well as the investigation of further particle contents could increase significance.

## 4. Discussion

The experimental results reveal that both static and fatigue strengths of an angle-ply GFRP wind power laminate can be significantly improved by nanomodification of the epoxy resin matrix. For a discrete design fatigue life, the modified composite containing 15 wt.% of boehmite particulates can bear about 10% higher cyclic maximum stresses, or, from the other perspective, fatigue life increases about 240% on average for a given load level. The maximum observed fatigue life enhancement is 270%. By means of optical monitoring and damage state evaluation, it could be shown that initiation of both matrix microcracking and interlaminar delamination is delayed by the particles. Furthermore, once initiated, damage growth rates are lower. As damage observations could only be conducted on the microscale, the underlying mechanisms (most likely plastic-void growth and particle debonding) of the fatigue strength increase could not be identified. Compared with results from literature (mainly 3- to 10-fold increases) [13,16,17,18,19,20,21], the achieved lifetime increase can be considered low but acceptable. It has to be pointed out that the effect on fatigue life crucially depends on particle type, constituents, laminate configuration and load ratio. Very high fatigue life increasements often are reached by means of particles which are problematic when infusing larger structures. In regards to the effect of load level, average fatigue life enhancements decrease with load level from 263% (at 70% UTS) to 220% (at 28% UTS) meaning a slight increase of the S-N curve slope. The reason for this could not be clarified, however, results in the literature range from slope increases [13,20,22,23] to slope decreases [17,21,27]. With regard to the significance of the results, it must be kept in mind that they refer to autoclave-processed laminates with only a low content of manufacturing-related voids. Therefore, findings may not simply be transferred to out-of-autoclave laminates with higher void contents.

The finding that the viscosity of the liquid nanocomposite can be effectively reduced by means of taurine particle surface modification without fatigue strength being affected, is of importance as well. It is the ability to keep viscosity low that enables the nanocomposite to be used in the infusion processes commonly employed in the wind power industry. However, as the viscosity of the taurine-modified nanocomposite remains higher than the viscosity of the unreinforced resin, process adjustments such as elevated mould temperatures and advanced through-the-thickness infusion might be necessary. The required infusion temperature, however, crucially depends on the infusion set-up and the flow-path.

With angle-ply configurations used in rotor blades, for example for the skin and the shear webs of the main spar [4,10], results are relevant for technical application. Typical damages such as parallel cracking of the skin [10], stress-whitening due to microcracks and interlaminar delamination [9] result from mechanisms that can be effectively delayed and slowed down by means of boehmite particles. Overall, matrix modification with low-cost taurine-modified boehmite particles is a promising approach for enhancing the fatigue resistance of wind turbine rotor blade materials.

## 5. Conclusions and Outlook

Comprehensive static and fatigue testing of GFRP angle ply specimens containing different amounts of taurine surface-modified boehmite nanoparticles were conducted. In addition to a fatigue life assessment, stiffness degradation and damage growth were investigated to clarify the impact of particle content on fatigue behaviour. Furthermore, for the particle content of 10 wt.%, the impact of both untreated and taurine-modified particles was characterised. Additionally to the regular cyclic tension-compression tests, the so-called Risitano fatigue limit (RFL) was determined for two laminate modifications, the reference laminate and the modification with the maximum particle content of 15 wt.%.

The main conclusions of this study are:

- Taurine surface modification of boehmite nanoparticles allows the viscosity of the nanocomposite to be significantly decreased. Using taurine-modified particles, composite laminates with high particle contents of up to 15 wt.% can therefore be fabricated via infusion without the typical infiltration issues.
- The static ultimate tensile stress (UTS) of the laminate increases with particle content.
- Fatigue life can be significantly increased by adding boehmite nanoparticles. For a particle content of 15 wt.%., maximum fatigue life enhancements of up to 270% were observed.
- Results indicate that fatigue life enhancement is load level-dependent. However, this finding could not be completely clarified.
- The taurine modification itself does not influence the fatigue strength.
- Initiation of the damage mechanisms (matrix cracking and delamination) is delayed and growth rates are smaller for the laminates containing boehmite nanoparticles. However, the mechanisms and their accumulation along the relative cycle number do not significantly change.
- Similar to the static strength, boehmite nanoparticles also increase the so-called Risitano fatigue limit.

Overall, the investigation reveals an improved material behaviour under cyclic loading for the nanoparticle-modified glass fibre reinforced plastic. At the same time, the viscosity of the particle-modified epoxy resin can be reduced by means of the taurine surface modification which makes it possible to process the nanocomposite via infusion. In this way, a sophisticated material with a high potential for rotor blades in wind turbines is presented.

In future investigations, further experiments have to be conducted to prove that fatigue life enhancements can also be achieved for out-of-autoclave laminates containing higher void contents. Regarding the operating conditions of wind energy rotor blades, the effect of the particle reinforcement also has to be investigated at non-ambient temperatures. Furthermore, even lower load levels have to be investigated to assess the results of the Risitano pseudo-fatigue limit estimation. Also, the observed load-level dependency of the life enhancement effect has to be clarified, for example by electron microscopic studies and analyses of load-level specific energy dissipation.

## Figures and Tables

**Table 1:** Overview of matrix modifications and static test results.

Matrix Modification	Number of Specimens	E-Modulus		Rupture Strain		Tensile Strength	
		$E_{x}/\mathrm{MPa}$	STD/MPa	${ɛ}_{x,ult}/\mathrm{MPa}$	STD/MPa	$\sigma_{ult}/\mathrm{MPa}$	STD/MPa
0 wt.% (Ref.)	7	13,021	236	3.20	0.26	94.8	2.14
5 wt.% (TM)	6	13,240	200	2.73	0.30	93.8	2.43
10 wt.%	6	14,846	103	2.67	0.20	99.6	3.45
10 wt.% (TM)	6	14,137	127	2.81	0.35	105.1	3.12
15 wt.% (TM)	6	15,099	119	2.84	0.32	107.2	1.94

**Table 2:** Overview of test parameters and fatigue test results.

LL	Load		Cycles per Block	Frequency	Specimens	Fatigue Cycles			Matrix Modification
	$\sigma_{a}$	$\sigma_{ult}/\sigma_{a}$	$N_{i}$	$f_{a}$	m	N	STD		
	MPa	-	-	Hz	-	-	-	%	
1	70	0.74	10	1	3	137	19	14	0 wt.% (Ref.)
		0.65			3	368	70	19	15 wt.%
2	55	0.58	100	3	3	2454	296	12	0 wt.% (Ref.)
		0.51			3	6157	256	4	15 wt.% (TM)
3	47	0.5	1000	3	5	7844	1386	18	0 wt.% (Ref.)
		-			3	15,160	689	5	0 wt.% (Ref.) (P)
		0.5			6	8305	734	9	5 wt.% (TM)
		0.47			4	13,159	1454	11	10 wt.%
		0.45			4	11,981	2157	18	10 wt.% (TM)
		0.44			4	18,414	1403	8	15 wt.% (TM)
		-			3	30,354	1795	6	15 wt.% (TM)(P)
4	40	0.42	5000	3	6	29,835	4490	15	0 wt.% (Ref.)
		0.37			4	66,054	10,445	16	15 wt.% (TM)
5	33	0.35	10,000	3	5	251,235	21,423	9	0 wt.% (Ref.)
		-			4	325,134	39,462	12	0 wt.% (Ref.) (P)
		0.35			7	235,973	39,799	17	5 wt.% (TM)
		0.33			4	295,235	55,958	19	10 wt.%
		0.31			4	317,201	57,003	18	10 wt.% (TM)
		0.31			4	529,167	67,316	13	15 wt.% (TM)
		-			3	631,415	64,046	10	15 wt.% (TM) (P)
6	28	0.3	50,000	6	4	1,486,201	264,921	18	0 wt.% (Ref.)
		0.26			4	3,624,331	1,028,596	28	15 wt.% (TM)

## Data Availability

Relevant data are included in the article.

## References

[1] Nijssen R.P.L. (2006). Fatigue Life Prediction and Strength Degradation of Wind Turbine Rotor Blade Composite.

[2] Katsaprakakis D.A., Papadakis N., Ntintakis I. (2021). A Comprehensive Analysis of Wind Turbine Blade Damage. Energies.

[3] Mishnaevsky L., Branner K., Nørgaard Petersen H., Beauson J., McGugan M., Sørensen B.F. (2017). Materials for Wind Turbine Blades: An Overview. Materials.

[4] Wessels S., Strobel M., van Wingerde A., Huessler I., Busmann H.-G. Improved fatigue design methods for offshore wind turbine rotor blades considering non-linear Goodman analysis combined with finite element analysis. Proceedings of the EWEC.

[5] Mandell J.F., Samborsky D.D. (1997). DOE/MSU Composite Material Fatigue Database: Test Methods, Materials and Analysis.

[6] Chen X., Eder M.A. (2020). A critical Review of Damage and Failure of Composite Wind Turbine Blade Structures. IOP Conf. Ser. Mater. Sci. Eng..

[7] Marin J.C., Barroso A., Paris F., Canas J. (2009). Study of fatigue damage in wind turbine blades. Eng. Fail. Anal..

[8] Choi S.-W., Farinholt K.M., Taylor S.G., Light-Marquez A., Park G. (2014). Damage Identification of Wind Turbine Blades Using Piezoelectric Transducers. Shock. Vib..

[9] Jüngert A. (2008). Damage Detection in Wind Turbine Blades Using two different Acoustic Techniques. e-J. Nondestruct. Test..

[10] Soerensen B.F., Joergensen E., Debel C.P., Jensen F.M., Jensen H.M., Jacobsen T., Halling K.M. (2004). Improved Design of Large Wind Turbine Blade of Fibre Composites Based on Studies of Scale Effects (Phase 1)—Summary Report.

[11] Karthikeyan R., Subbiah R., Ranganathan N., Bensingh J., Kader A., Nayak S. (2020). A review on fatigue damages in the wind turbines: Challenges in determining and reducing fatigue failures in wind turbine blades. Wind. Eng..

[12] Castro O. (2018). Fatigue Strength of Composite Wind Turbine Blade Structures. DTU Wind. Energy.

[13] Tate J.S., Akinola A.T., Espinoza S., Gaikwad S., Vasudevan D.K.K., Sprenger S., Kumar K. (2018). Tension–tension fatigue performance and stiffness degradation of nanosilica-modified glass fiber-reinforced composites. J. Compos. Mater..

[14] Rafiee M., Rad S.H., Nitzsche F., Laliberte J., Labrosse M.R. (2020). Significant fatigue life enhancement in Mulitscale Doubly-Modified Fiber/Epoxy Nanocomposites with Graphene Nanoplatelets and Reduced-Graphene Oxide. Polymers.

[15] Phong N.T., Gabr M.H., Anh L.H., Duc V.M., Betti A., Okubo K., Chuong B., Fujii T. (2013). Improved fracture toughness and fatigue life of carbon fiber reinforced epoxy composite due to the incorporation of rubber nanoparticles. J. Mater. Sci..

[16] Fenner J.S., Daniel I.M. (2014). Hybrid nanoreinforced carbon/epoxy composites for enhanced damage tolerance and fatigue life. Compos. Part A.

[17] Makeev A., Bakis C., Strauch E., Chris M., Holemans P., Miller G., Nguyen D., Spencer D., Patz N. (2015). Advanced Composite Materials Technology for Rotorcraft through the Use of Nanoadditives. J. Am. Helicopter Soc..

[18] Jagannathan N., Bojja R., Manjunatha C.M., Taylor A.C., Kinloch A.J. (2013). Fatigue Behaviour of a Hybrid Particle Modified Fiberglass/Epoxy Composite under a Helicopter Spectrum Load Sequence. Adv. Compos. Lett..

[19] Megahed M., El-Wafa Megahed A.A., Agwa M.A. (2018). Mechanical properties of on/off-axis loading for hybrid glass fiber reinforced epoxy filled with silica and carbon black nanoparticles. Mater. Technol..

[20] Manjunatha C.M., Taylor A.C., Kinloch A.J., Sprenger S. (2010). The tensile fatigue behavior of a silica nanoparticle-modified glass fibre reinforced epoxy composite. Compos. Sci. Technol..

[21] Manjunatha C.M., Sprenger S., Taylor A.C., Kinloch A.J. (2010). The Tensile Fatigue Behavior of a Glass-fiber Reinforced Plastic Composite Using a Hybrid-toughened Epoxy Matrix. J. Compos. Mater..

[22] Knoll J.B., Riecken B.T., Kosmann N., Chandrasekaran S., Schulte K., Fiedler B. (2014). The effect of carbon nanoparticles on the fatigue performance of carbon fibre reinforced epoxy. Compos. Part A.

[23] Bourchak M., Algarni A., Khan A., Khashaba U. (2018). Effect of SWCNTs and graphene on the fatigue behavior of antisymmetric GFRP laminate. Compos. Sci. Technol..

[24] Manjunatha C.M., Bojja R., Jagannathan N., Kinloch A.J., Taylor A.C. (2013). Enhanced fatigue behavior of a glass fiber reinforced hybrid particles modified epoxy nanocomposite under WISPERX spectrum load sequence. Int. J. Fatigue.

[25] Ngah S.A., Taylor A.C. (2018). Fracture behavior of rubber- and silica nanoparticle-toughened glass fibre composites under static and fatigue loading. Compos. Part A.

[26] Fathy A., Shaker A., Abdel Hamid M., Megahed A.A. (2017). The effects of nano-silica/nano-alumina on fatigue behavior of glass fiber-reinforced epoxy composites. J. Compos. Mater..

[27] Kamble M., Lakhnot A.S., Bartolucci S.F., Littlefield A.G., Picu C.R., Koratkar N. (2020). Improvement in fatigue life of carbon fibre reinforced polymer composites via a Nano-Silica Modified Matrix. Carbon.

[28] Jux M., Fankhänel J., Daum B., Mahrholz T., Sinapius M., Rolfes R. (2018). Mechanical properties of epoxy/boehmite nanocomposites in dependency of mass fraction and surface modification—An experimental and numerical approach. Polymer.

[29] Jux M., Finke B., Mahrholz T., Sinapius M., Kwade A., Schilde C. (2017). Effects of Al(OH)O nanoparticle agglomerate size in epoxy resin on tension, bending, and fracture properties. J. Nanoparticle Res..

[30] Exner W., Arlt C., Mahrholz T., Riedel U., Sinapius M. (2012). Nanoparticles with various surface modifications as functionalized crosslinking agents for composite resin materials. Compos. Sci. Technol..

[31] Arlt C., Exner W., Riedel U., Sturm H., Sinapius M., Ziegmann G. (2021). Sinapius, J.M. Nanoscaled Boehmites’ Modes of Action in a Polymer and Its Carbon Fiber Reinforced Plastic. Acting Principles of Nano-Scaled Matrix Additives for Composite Structures.

[32] Jux M. (2021). Einfluss der Grenzflächen Nanoskaliger Matrixadditive Auf das Schlagzähigkeitsverhalten von Faserverbunden, Dissertation.

[33] LaRosa G., Risitano A. (2000). Thermographic methodology for rapid determination of the fatigue limit of materials and mechanical components. Int. J. Fatigue.

[34] Deutsches Institut für Normung (1997). Kunststoffe—Bestimmung der Zugeigenschaften—Teil 4: Prüfbedingungen für Isotrop und Anisotrop Faserverstärkte Kunststoffverbundwerkstoffe/Plastics—Determination of Tensile Properties—Part 4: Test Conditions for Isotropic and Anisotropic Fibre-Reinforced Plastic Composites.

[35] Bryan H. (2003). Fatigue Composite Materials.

[36] Schneider C.R.A., Maddox S.J. (2003). Best Practice Guide on Statistical Analysis of Fatigue Data.

[37] Luong M.P. (1998). Fatigue Limit Evaluation of Metals using an Infrared Thermographic Technique. Mech. Mater..

[38] Krapez J.C., Pacou D. Thermography detection of damage initiation during fatigue tests. Proceedings of the Thermosense XXIV, Proc. SPIE 4710.

[39] Quaresimin M. Fatigue of woven composite laminates under tensile and compressive loading. Proceedings of the European Conference on Composite Materials, ECCM 10.

[40] Colombo C., Libonati F., Pezzani F., Salerno A., Vergani L. (2011). Fatigue Behaviour of a GFRP laminate by thermographic measurements. Procedia Eng..

[41] Gornet L., Westphal O., Burtin C., Bailleul J.L., Rozycki P. (2013). Rapid Determination of the High Cycle Fatigue Limit Curve of Carbon Fiber Epoxy Matrix Composite Laminates by Thermography Methodology: Tests and Finite Element Simulations. Procedia Eng..

[42] Jegou L., Marco Y., Le Saux V., Calloch S. (2013). Fast prediction of the Wöhler curve from heat build-up measurements on Short Fiber Reinforced Plastic. Int. J. Fatigue.

[43] Montesano J., Fawaz Z., Bougherara H. (2013). Use of infrared thermography to investigate the fatigue behavior of a carbon fiber reinforced polymer composite. Compos. Struct..

[44] Tang Y., Ye L., Zhang Z., Friedrich K. (2013). Interlaminar fracture toughness and CAI strength of fibre-reinforced composites with nanoparticles-A review. Compos. Sci. Technol..

[45] (2003). ISO International Standard: ISO13003—Fibre-Reinforced Plastics—Determination of Fatigue Properties under Cyclic Loading Conditions.

[46] Reifsnider K.L. (1991). Fatigue of Composite Materials.

[47] Talreja R., Singh C.V. (2012). Damage and Failure of Composite Materials.

[48] Adam T.J., Horst P. (2017). Fatigue damage and fatigue limits of a GFRP angle-ply laminate tested under very high cycle fatigue loading. Int. J. Fatigue.

